# Transcription and chromatin regulation by TAF4b during cellular quiescence of developing prospermatogonia

**DOI:** 10.3389/fcell.2023.1270408

**Published:** 2023-10-12

**Authors:** Megan A. Gura, Myles A. Bartholomew, Kimberly M. Abt, Soňa Relovská, Kimberly A. Seymour, Richard N. Freiman

**Affiliations:** ^1^ MCB Graduate Program, Providence, RI, United States; ^2^ Department of Molecular Biology, Cell Biology, and Biochemistry, Brown University, Providence, RI, United States

**Keywords:** transcription, spermatogenesis, prospermatogonia, quiescence, TAF4B, spermatogonial stem cells, male fertility

## Abstract

Prospermatogonia (ProSpg) link the embryonic development of male primordial germ cells to the healthy establishment of postnatal spermatogonia and spermatogonial stem cells. While these spermatogenic precursor cells undergo the characteristic transitions of cycling and quiescence, the transcriptional events underlying these developmental hallmarks remain unknown. Here, we investigated the expression and function of TBP-associated factor 4b (*Taf4b*) in the timely development of quiescent mouse ProSpg using an integration of gene expression profiling and chromatin mapping. We find that *Taf4b* mRNA expression is elevated during the transition of mitotic-to-quiescent ProSpg and *Taf4b-*deficient ProSpg are delayed in their entry into quiescence. Gene ontology, protein network analysis, and chromatin mapping demonstrate that TAF4b is a direct and indirect regulator of chromatin and cell cycle-related gene expression programs during ProSpg quiescence. Further validation of these cell cycle mRNA changes due to the loss of TAF4b was accomplished via immunostaining for proliferating cell nuclear antigen (PCNA). Together, these data indicate that TAF4b is a key transcriptional regulator of the chromatin and quiescent state of the developing mammalian spermatogenic precursor lineage.

## Introduction

Male fertility is dependent on a highly regulated series of developmental events that begin with primordial germ cell (PGC) specification in early fetal development and end with the exhaustion of an adult unipotent spermatogonial stem cell (SSC) population during old age (De Rooij and Grootegoed, 1998; Oatley and Brinster, 2008; Phillips et al., 2010). In mammals, PGC specification is achieved via BMP signals secreted from a small group of dorsally localized extra-embryonic cells to the posterior epiblast. This then induces the expression of the master transcription regulators PRDM1, PRDM14, and TFAP2C (Ohinata et al., 2005; Yamaji et al., 2008; Weber et al., 2010). While more heterogeneous in nature, several transcription factors mark and/or support adult SSC identity, including ID4, RHOX10, PAX7, and SALL4 (Oatley et al., 2011; Gassei and Orwig, 2013; Aloisio et al., 2014; Song et al., 2016; Fayomi and Orwig, 2018; Green et al., 2018; Shami et al., 2020). Prospermatogonia (ProSpg, also called ‘gonocytes’) are male germ cells that have differentiated past PGC specification and have the potential to differentiate into adult spermatogonia (Spg) or become part of the SSC pool which provides the long-term renewing capabilities of the testis. Several critical molecular events occur during ProSpg development. First, epigenetic marks that were erased in PGCs are re-established in ProSpg. Second, an initial pool of unipotent SSCs is thought to arise from the heterogeneous pool of developing ProSpg during this developmental window (Kubo et al., 2015; Hill et al., 2018). These events are necessary to initiate spermatogenesis in a timely manner and maintain it properly throughout adulthood. However, the underlying gene expression networks that correctly time and integrate these complex events are currently unknown.

Sequence-specific transcription factors are thought to recruit RNA Polymerase II (RNAPII) to specific core promoters via coactivators and the general transcription machinery. How the regulation of RNAPII transcription is achieved in ProSpg and how it is integrated with epigenetic reprogramming of the male germline genome at these developmental stages is unknown. We have identified a unique germ cell-enriched subunit of the general transcription factor TFIID, called TAF4b, that is required for proper long-term spermatogenesis in the mouse. Male mice lacking TAF4b become infertile after undergoing a first round of spermatogenesis; however, spermatogenesis can be re-established in the *Taf4b*-deficient testis following transplant of wildtype SSCs reflecting a functional adult SSC niche (Falender et al., 2005). More recent characterization of spermatogenesis in our *Taf4b*-deficient mice revealed that TAF4b is required for embryonic male germ cell development and helps regulate the delicate balance of SSC self-renewal and differentiation (Lovasco et al., 2015). In addition to studies of TAF4b in mice, human males who express a truncated version of the TAF4b protein are infertile owing to progressive azoospermia (Ayhan et al., 2014), and single nucleotide polymorphisms in human TAF4b have been linked to nonobstructive azoospermia (Xi et al., 2020). Thus, deciphering the developmental and molecular mechanisms of TAF4b in the mouse can improve our understanding and model important aspects of male reproductive development and fertility in humans.

To contextualize the progressive loss of *Taf4b*-deficient male germ cells during embryogenesis, we examined the spatial and temporal expression of TAF4b during embryonic testis development. We analyzed published RNA-seq data sets derived from E9.5-E18.5 *Oct4*-eGFP mice, in which germ cells were separated from somatic cell populations through fluorescence-activated cell sorting (FACS (Gura et al., 2020)). We determined that levels of *Taf4b* mRNA become progressively and significantly elevated in a germ cell-specific manner from embryonic day (E) 11.5 to E18.5. Germ cell-enriched TAF4b protein expression at E13.5 suggests it plays an important germ cell-specific role during ProSpg development (Gura et al., 2020). Mitotic stage (M) ProSpg are proliferative between E13.5 and E16.5, at which they then progress to transitional 1 (T1) ProSpg and enter a period of cellular quiescence. Soon after birth, T1 ProSpg become transitional 2 (T2) ProSpg by reentering the cell cycle until they become Spg at approximately postnatal day (P) 8 (McCarrey, 2013). Recent studies by Law et al., 2019, suggest that a subpopulation of T1 ProSpg acquires high *Id4* expression, which marks a potential initial pool of SSCs (Law et al., 2019). However, the transcriptional logic underlying these ProSpg transitions remains unknown.

Here, we tested if and how TAF4b regulates critical transcriptional programs and cell states during early ProSpg development. First, we examined *Taf4b* expression in published single-cell (sc) RNA-seq data and confirmed it is enriched in quiescent T1 ProSpg. Next, we performed bulk RNA-seq on *Taf4b*-deficient quiescent ProSpg at E16.5 to reveal cell cycle and chromatin structure as top gene ontology (GO) categories modulated in the absence of TAF4b. For genomic mapping of TAF4b localization in T1 ProSpg, we employed cleavage under targets and release using nuclease (CUT&RUN), which identified 617 TAF4b peaks just upstream of numerous transcription start sites (TSSs). Distinctive GO categories from the CUT&RUN included RNA processing, chromatin modification, and cell cycle regulation. The most consistent DNA motifs within the TAF4b-bound promoters included Sp/Klf family and NFY binding sites; a remarkable similarity to our findings in embryonic mouse oocytes, and for the first time linking these ubiquitously-expressed transcription factors to the precise regulation of ProSpg development (Gura et al., 2022). At the cellular level, we determined that *Taf4b*-deficient ProSpg are delayed in their entry to quiescence, noting significant perturbation from controls at E14.5 and that these cells also display key cell cycle protein changes during ProSpg quiescence. Together, these data suggest that TAF4b is a dynamic and vital integrator of male germline transcription and chromatin states that promotes the timely quiescence of ProSpg required for further mammalian spermatogenic development.

## Materials and methods

### Mice

Mice, homozygous for an *Oct4-eGFP* transgene (The Jackson Laboratory: B6; 129S4- *Pou5f1* ^*tm2Jae*^/J), were mated for CUT&RUN collections. Mice, homozygous for an *Oct4-eGFP* transgene (The Jackson Laboratory: B6; 129S4-*Pou5f1* ^*tm2Jae*^/J) and heterozygous for the *Taf4b*-deficiency mutation (in exon 12 of the 15 total exons of the *Taf4b* gene that disrupts the endogenous *Taf4b* gene), were mated for mRNA collections. Timed matings were estimated to begin at day 0.5 by evidence of a copulatory plug. The sex of the embryos was identified by confirming the presence or absence of testicular cords. Genomic DNA, isolated from tail biopsies using QIAGEN DNeasy Blood & Tissue Kits (Cat #: 69506), was used for PCR genotyping assays.

All animal protocols were reviewed and approved by Brown University Institutional Animal Care and Use Committee and were performed in accordance with the National Institutes of Health Guide for the Care and Use of Laboratory Animals. Gonads were dissected out of embryos into cold PBS.

### Immunofluorescence

Testes were gathered from embryonic mice (E13.5-E18.5), briefly fixed with 10% formalin and paraffin-embedded. The Molecular Pathology Core at Brown University completed tissue preparation and performed sectioning. After the serial sections were prepared, antigen retrieval was performed using a 0.1% antigen unmasking solution (Vector Laboratories, H-3300) in a steamer for 20 min. Samples were then permeabilized in 0.1% sodium citrate and Triton X-100 for 10 min, washed with 0.1% PBST (Triton X-100), and incubated in blocking buffer (PBS with 3% goat serum, 1% BSA, and 0.1% Triton X-100) for 1 hour. This was then followed by primary antibody incubation for 24 h at 4°C prepared in the blocking buffer. The samples were then washed in PBST, incubated with secondary antibody for 1 h at 37°C, washed in PBST, and mounted in DAPI-containing Vectashield Mounting Medium (Vector Laboratories H-1200, Burlingame, CA). Primary antibodies used were rat anti-TRA98 (1:100, abcam, ab82527), anti-KI67 rabbit mAb (1:100, Cell Signaling Technology D3B5, 9129S) and anti-PCNA (1:100, Cell Signaling Technology, 13110T). For immunofluorescence, the secondary antibodies used were Alexa Fluor 555-conjugated anti-rat IgG (1:500; abcam ab150158) and Alexa Fluor 488-conjugated anti-rabbit IgG (1:500; abcam ab150081). Images were all acquired at the same exposures and received the same immunofluorescence preparation. Visualization and counting were completed via blinded assessment of TRA98-expressing cells colocalized with MKI67 on a Zeiss Axio Imager M1 and Zeis software. A two-way ANOVA was used to determine significance using GraphPad Prism 9 version 9.3.1. for MacOS (GraphPad Software, Boston, Massachusetts United States, www.graphpad.com) with an n of 3-5 mice.

### Embryonic gonad dissociation and fluorescence-activated cell sorting

To dissociate gonadal tissue into a single-cell suspension, embryonic gonads were harvested and placed in 0.25% Trypsin/EDTA and incubated at 37°C for 15 and 25 min for E14.5 and E16.5 gonads, respectively, as previously described (Gura et al., 2020). Eppendorf tubes were flicked to dissociate tissue halfway through and again at the end of the incubation. Trypsin was neutralized with FBS. Cells were pelleted at 1,500 RPM for 5 min, the supernatant was removed, and cells were resuspended in 100 μL PBS. The cell suspension was strained through a 35 μm mesh cap into a FACS tube (Gibco # 352235). Propidium iodide (1:500) was added to the cell suspension as a live/dead distinguishing stain. Fluorescence-activated cell sorting (FACS) was performed using a Becton Dickinson FACSAria III in the Flow Cytometry and Cell Sorting Core Facility at Brown University. A negative control of a non-transgenic mouse gonad was used for each experiment to establish an appropriate GFP signal baseline. Dead cells were discarded and the remaining cells were sorted into GFP^+^ and GFP^−^ samples in PBS at 4°C for each embryo.

For RNA-seq analysis, GFP^+^ cells from each individual embryo were kept in separate tubes and were then spun down at 1,500 RPM for 5 min, had PBS removed, and were then resuspended in Trizol (ThermoFisher # 1556026). If samples had roughly less than 50 µL of PBS in the tube, Trizol was added immediately. The number of cells for each sample can be found in Supplementary Table S1. Samples were stored at −80°C.

For CUT&RUN, germ cells from all the gonads were pooled prior to FACS. Sorted germ cells were then spun down at 1,500 RPM for 5 min and were resuspended in 300 µL of PBS, then split into three Eppendorf tubes. These three tubes of germ cells were then used for CUT&RUN. The number of cells for each sample were as follows: Replicate 1 germ cell samples had approximately 56,000 cells per tube (obtained from 22 embryos) and Replicate 2 germ cell samples had approximately 131,000 cells per tube (obtained from 28 embryos).

### Single cell RNA-seq data analysis

All computational scripts regarding single cell RNA-seq (scRNA-seq) used in this publication are available to the public: https://github.com/mg859337/Gura_et_al._TAF4b_male_transcription. SRP194420, SRP158811, and SRP178196 were downloaded from NCBI SRA onto Brown University’s high-performance computing cluster at the Center for Computation and Visualization. We used Law et al. and Nguyen et al., which enriched for germ cells via FACS sorting (Law et al., 2019; Nguyen et al., 2020). We then combined the data with Tan et al., who collected the whole testis, thus establishing our comprehensive observation window from E12.5 to P7 (Tan et al., 2020). The fastq files were aligned using Cell Ranger (v 6.0.0) count and then aggregated using Cell Ranger aggr. The cloupe file created from Cell Ranger aggr was used as input for Loupe Cell Browser (v 5.0). We selected the germ cells by first assessing the log-normalized expression of germ cell marker *Dazl* within the complete 71,584 cell data set. Then, using unbiased clustering, we eliminated somatic cell clusters based on their mean *Dazl* expression being less than 0.85. The data set underwent further quality control and filtering based on fitting the metrics of 12.5–15.8 log2-normalized unique molecular identifiers, 10.6–12.8 log2-normalized features, and lastly, less than 24% mitochondrial transcript ratio (Quatredeniers et al., 2023).

### RNA-sequencing

Embryonic germ cells resuspended in Trizol were shipped to GENEWIZ (GENEWIZ Inc., NJ) on dry ice. Sample RNA extraction, sample QC, library preparation, sequencing, and initial bioinformatics were done at GENEWIZ. RNA was extracted following the Trizol Reagent User Guide (ThermoFisher Scientific). Glycogen was added (1 μL, 10 mg/mL) to the supernatant to increase RNA recovery. RNA was quantified using Qubit 2.0 Fluorometer (Life Technologies, Carlsbad, CA, United States) and RNA integrity was checked with TapeStation (Agilent Technologies, Palo Alto, CA, United States) to see if the concentration met the requirements.

SMART-Seq v4 Ultra Low Input Kit for Sequencing was used for full-length cDNA synthesis and amplification (Clontech, Mountain View, CA), and Illumina Nextera XT Library Preparation Kit was used for library preparation. The sequencing libraries were multiplexed and clustered on a lane of a flowcell. After clustering, the flowcell was loaded onto an Illumina HiSeq 4000 according to manufacturer’s instructions. The samples were sequenced using a 2 × 150 Paired End (PE) configuration. Image analysis and base calling were conducted by the HiSeq Control Software (HCS) on the HiSeq instrument. Raw sequence data (.bcl files) generated from Illumina HiSeq were converted into fastq files and de-multiplexed using bcl2fastq (v 2.17). One mismatch was allowed for index sequence identification.

### RNA-seq data analysis

All computational scripts regarding RNA-seq used in this publication are available to the public: https://github.com/mg859337/Gura_et_al._TAF4b_male_transcription. All raw fastq files were initially processed on Brown University’s high-performance computing cluster. Reads were quality-trimmed and had adapters removed using Trim Galore! (v 0.5.0) with the parameters–nextera -q 10. Samples before and after trimming were analyzed using FastQC (v 0.11.5) for quality and then aligned to the Ensembl GRCm38 using HiSat2 (v 2.1.0) (Andrews, 2010; Pertea et al., 2016). Resulting sam files were converted to bam files using Samtools (v 1.9) (Li et al., 2009).

To obtain TPMs for each sample, StringTie (v 1.3.3b) was used with the optional parameters -A and -e. A gtf file for each sample was downloaded and, using RStudio (R v 4.0.2), TPMs of all samples were aggregated into one comma separated (csv) file using a custom R script. To create interactive Microsoft Excel files for exploring the TPMs of each dataset: the csv of aggregated TPMs was saved as an Excel spreadsheet (Supplementary Table S2). Colored tabs were added to set up different comparisons, and a flexible Excel function was created to adjust to gene name inputs. To explore the Excel files, please find the appropriate tab (named “Quick_Calc”) and type in the gene name of interest into the highlighted yellow boxes.

To obtain count tables, featurecounts (Subread v 1.6.2) was used (Liao et al., 2014). Metadata files for each dataset were created manually in Excel and saved as a csv. These count tables were used to create PCA plots by variance-stabilizing transformation (vst) of the data in DESeq2 (v 1.22.2) and plotting by ggplot2 (v 3.1.0) (Love et al., 2014; Wickham, 2016). DESeq2 was also used for differential gene expression analysis, where count tables and metadata files were used as input. We accounted for the litter batch effect in our mouse germ cells by setting it as a batch parameter in DESeq2. For the volcano plot, the output of DESeq2 was used and plotted using ggplot2. DEG lists were used for ClusterProfiler (v 3.16.1) input to create dotplots of significantly enriched gene ontology (GO) categories for all DEGs. The highest-confidence protein-protein interactions were identified using STRING, with unconnected proteins not shown in the images (Szklarczyk et al., 2019). Net plots were constructed using Bioconductor packages in R after normalization and QC. The top three GO processes category nets for both Up and Downregulated DEGs were cross-referenced with the TAF4b-bound gene TSS list.

### CUT&RUN

The CUT&RUN performed in E16.5 germ cells followed the protocol in Hainer and Fazzio, 2019 (Hainer and Fazzio, 2019). CUT&RUN antibodies were as follows: polyclonal rabbit TAF4b (as previously described (Grive et al., 2016))*,* monoclonal rabbit H3K4me3 (EMD Millipore # 05-745R), rabbit IgG (ThermoFisher # 02–6102), pA-MNase was a generous gift from Dr. Thomas Fazzio, UMASS Med. For library preparation, the KAPA HyperPrep Kit (Roche Cat. No 07962363001) was used with New England Biolabs NEBNext Multiplex Oligos for Illumina (NEB #E7335). After library amplification through PCR, libraries were size-selected through gel extraction (∼150–650 bp) and cleaned up using the QIAGEN QIAquick Gel Extraction Kit (Cat. # 28704). CUT&RUN libraries in EB buffer were shipped to GENEWIZ (GENEWIZ Inc., NJ) on dry ice. Sample QC, sequencing, and initial bioinformatics were done at GENEWIZ.

The sequencing libraries were validated on the Agilent TapeStation (Agilent Technologies, Palo Alto, CA, United States), and quantified by using Qubit 2.0 Fluorometer (Invitrogen, Carlsbad, CA) as well as by quantitative PCR (KAPA Biosystems, Wilmington, MA, United States). The sequencing libraries were clustered on flowcells. After clustering, the flowcells were loaded onto the Illumina HiSeq instrument (4,000 or equivalent) according to manufacturer’s instructions. The samples were sequenced using a 2 × 150bp Paired End (PE) configuration. Raw sequence data (.bcl files) generated from Illumina HiSeq were converted into fastq files and de-multiplexed using bcl2fastq (v. 2.20). One mismatch was allowed for index sequence identification.

### CUT&RUN data analysis

All computational scripts regarding CUT&RUN data analysis used in this publication are available at: https://github.com/mg859337/Gura_et_al._TAF4b_male_transcription and based on other CUT&RUN publications (Hainer and Fazzio, 2019). All raw fastq files were initially processed on Brown University’s high-performance computing cluster. Reads were quality-trimmed and had adapters removed using Trim Galore! (v 0.5.0) with the parameter -q 10 (https://www.bioinformatics.babraham.ac.uk/projects/trim_galore/). Samples before and after trimming were analyzed using FastQC (v 0.11.5) for quality and then aligned to the Ensembl GRCm39 using Bowtie2 (v 2.3.0). Fastq screen (v 0.13.0) was used to determine the percentage of reads uniquely mapped to the mouse genome in comparison to other species. Resulting sam files were converted to bam files, then unmapped, duplicated reads, and low quality mapped were removed using Samtools (v 1.9). Resulting bam files were split into size classes using a Unix script. For calling peaks, annotating peaks, and identifying coverage around TSSs, Homer (v 4.10) was used (Heinz et al., 2010). For gene track visualization, the final bam file before splitting into size classes was used as input to Integrative Genomics Viewer (IGV) (Robinson et al., 2011). A custom genome was created using a genome fasta and gtf file for Ensembl GRCm39.

Pie charts were created using data from Homer output and Venn diagrams were created using BioVenn. Dotplots of Promoter-TSS peaks were made using ClusterProfiler. TSS plots were created using the “tss” function of Homer and plotted using Microsoft Excel. All plots produced in RStudio were saved as an EPS file type and then opened in Adobe Illustrator in order to export a high-quality JPEG image. Excel file of the composite CUT&RUN replicates is presented as several unique tabs in Supplementary Table S3.

## Results

### scRNA-seq reveals peak *Taf4b* mRNA expression coincides with ProSpg quiescence

**FIGURE 1:**
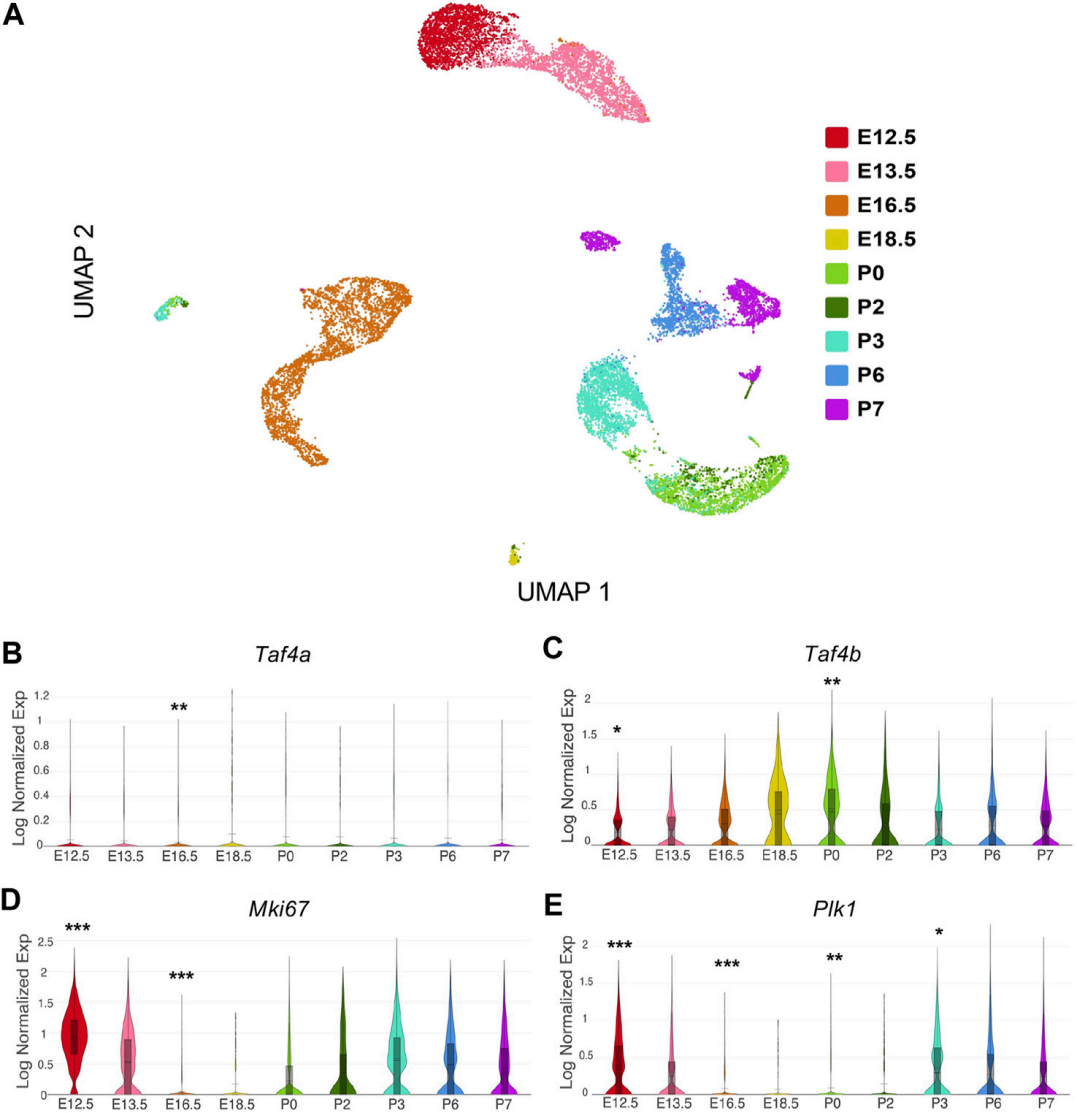
Characterizing Taf4b mRNA expression across male germ cell development in mouse. **(A)** UMAP of E12.5-P7 germ cells colored by time point. **(B–E)** Expression of *Taf4a, Taf4b, Mki67,* and *Plk1* across the developmental time course. *p*-values are denoted (*** = *p*< 0.001, ** = *p*< 0.01, * = *p*< 0.05) and are adjusted using the Benjamini-Hochberg correction method for multiple tests.

Analyzing single-cell RNA-seq data allowed us to examine the gene expression within ProSpg at finer resolution, which we reprocessed from published publicly available data sets (Tan et al., 2020; Gura et al., 2022; Quatredeniers et al., 2023). In our analysis, we examined the expression profile of *Taf4b* over time to evaluate what genes and biological processes are possibly modulated concurrently or downstream of TAF4b expression. After applying our computational workflow (see Methods), the complete data set consisted of 17,310 germ cells in our uniform manifold approximation and projection (UMAP) (Figure 1A). We then assessed the expression of *Taf4b* compared to its paralog *Taf4a*, which is ubiquitously expressed in somatic cells*.* Across all time points, *Taf4a* mRNA expression was notably lower than *Taf4b* mRNA expression (Figures 1B,C). When observing the expression of *Taf4b* mRNA over the E12.5-P7 time course, we detected that *Taf4b* expression peaks at P0 (Figure 1C). The dynamic range of *Taf4b*-expressing cells across time suggests *Taf4b* may act more akin to a dimmer switch than on/off regulation. Because previous experiments demonstrated that the *Taf4b*-deficient gonad exhibited changes in germ cell proliferation, we compared this newly characterized *Taf4b* expression profile to known proliferation markers, such as *Mki67* (Sun and Kaufman, 2018). We saw an inverse expression pattern of *Mki67* and *Taf4b,* where the significantly reduced expression of *Mki67* between E16.5-P0 points to the characteristic quiescent period of T1 ProSpg (Figure 1D). The absence of *Mki67* transcripts helps illustrate quiescence because its mRNA is expressed during S phase up until mitotic exit. *Plk1* mRNA, another marker of cell proliferation, is decreased similarly from E16.5 to P2 (Figure 1E) (Liu et al., 2017). The combined decreased markers of proliferation and increased *Taf4b* expression led us to hypothesize that *Taf4b* may play a role in the cell cycle transitions during ProSpg development.

### Global ProSpg RNA-seq reveals cell cycle and chromatin gene expression affected by TAF4b

**FIGURE 2:**
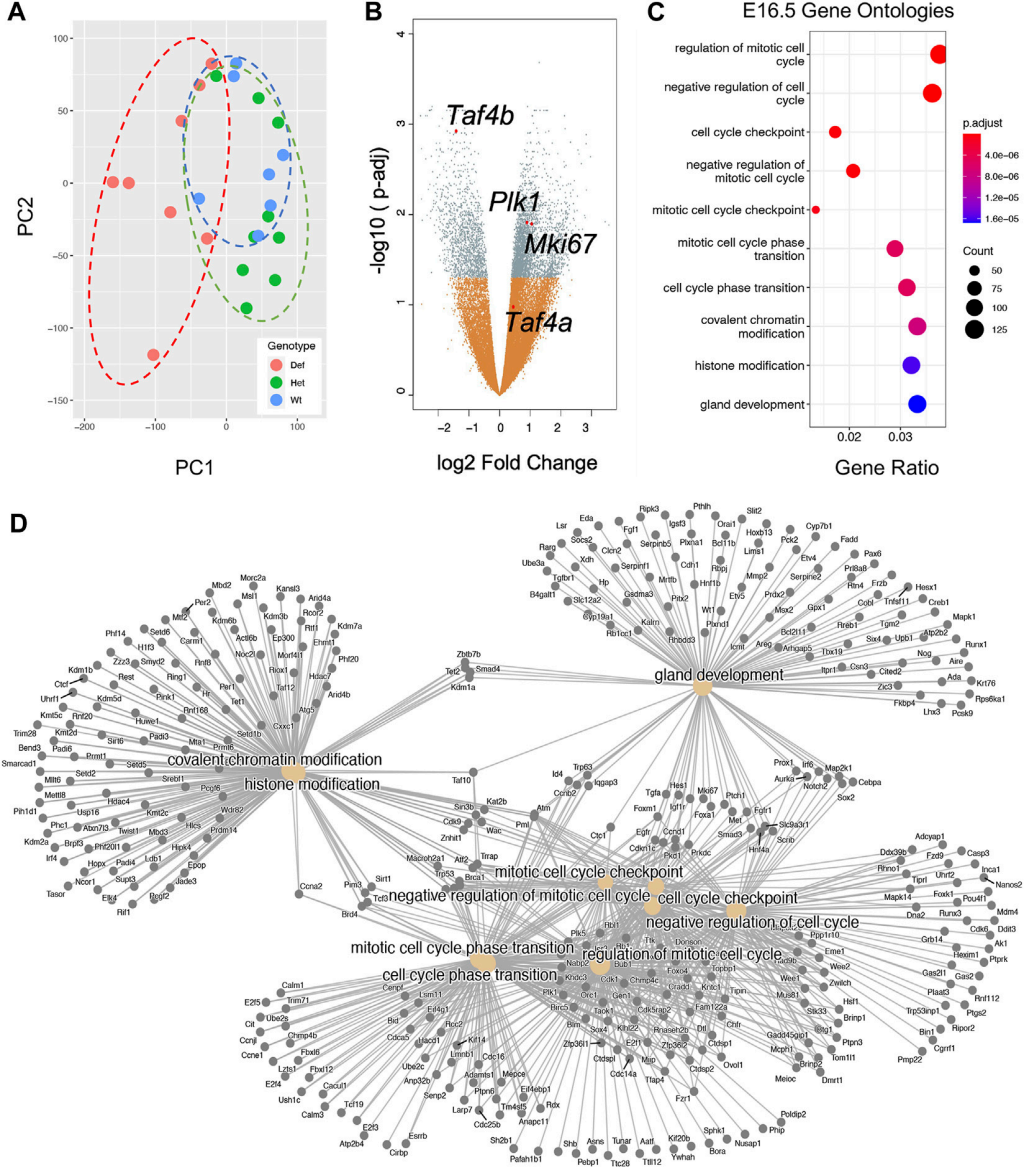
E16.5 ProSpg RNA-seq experiment reveals significant regulation of mitotic gene programs. **(A)** PCA plot of the E16.5 samples labeled based on *Taf4b* genotype as indicated by color and dotted lines. **(B)** Volcano plot of genes; the 4551 significant genes (p-adj <0.05) are labeled in grey, DEGs of interest plus *Taf4b* are specified and nonsignificant gene changes are labeled in orange. **(C)** Dotplot of GO biological process analysis of all 3820 protein-coding DEGs from **(B)**. **(D)** Category netplot showing the relationships between the top ten GO biological processes from **(C)** and the individual DEGs within each ontology.

We were intrigued to observe a clear increase in *Taf4b* mRNA during the ProSpg transition to quiescence. To better understand the potential function of TAF4b during this transition, we compared global mRNA differences between *Taf4b*-wildtype, -heterozygous, and -deficient ProSpg at E16.5. We first generated mouse embryos that were transgenic for *Oct4*-eGFP and either wildtype, heterozygous, or deficient for *Taf4b*, then sorted for GFP^+^ germ cells from the male embryonic testis. This provided germ cell-isolated samples from all three genotypes to perform bulk RNA-seq. Our ProSpg RNA-seq samples consisted of 7 *Taf4b*-wildtype, 9 *Taf4b*-heterozygous, and 8 *Taf4b*-deficient E16.5 embryos from six different litters (Supplementary Table S1). Although *Taf4b-*heterozygous male mice do not display overt signs of impaired spermatogenesis (Falender et al., 2005), including them as a unique hypomorphic and genotypic group was important. A PCA plot of these samples indicates that samples cluster primarily by genotype. While *Taf4b*-wildtype (WT) replicates display the tightest clustering, they do overlap with the *Taf4b*-heterozygous (Het) replicates, indicating a shared transcriptional profile (Figure 2A). This was expected as both genotypes are fertile. In contrast, the *Taf4b*-deficient (Def) replicates were much more dispersed and non-overlapping with the other two genotypes, suggesting large-scale transcriptional dysregulation. There were three Def samples that clustered more closely with the WT and Het samples. However, we completed the downstream analysis with all replicates because there were subtle but statistically significant transcriptional differences in these three samples compared to WT/HET. Overall, we detected 4551 DEGs between the *Taf4b*-wildtype and -deficient ProSpg; 3820 were defined as protein-coding with an adjusted *p*-value <0.05 (Figure 2B). Some genes noted to be dysregulated were *Mki67* and *Plk1* (Liu et al., 2017; Sun and Kaufman, 2018). These markers of proliferation should decrease during this quiescent time point but are significantly upregulated in the *Taf4b*-deficient ProSpg (Figure 2B). Using gene ontology (GO) of the DEGs, we determined which biological processes are disrupted in the absence of *Taf4b*. This GO analysis indicated cell cycle regulation is the primary disruption driven by *Taf4b* ablation, followed by chromatin modification and gland development. (Figures 2C,D).

**FIGURE 3:**
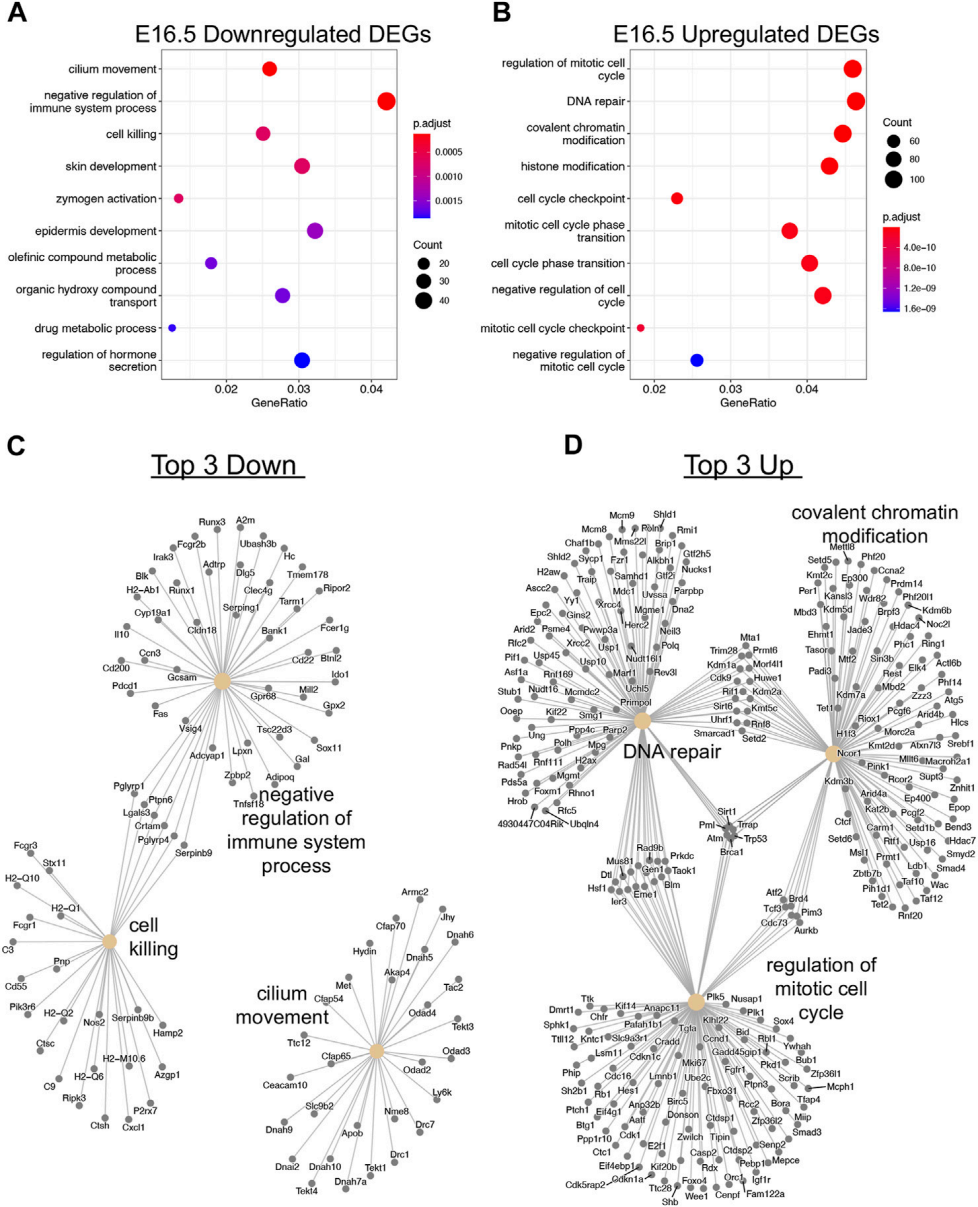
Transcriptional dysregulation of cell cycle and chromatin modification genes. **(A)** Dotplot of GO biological process analysis of 1,259 protein-coding Down DEGs. **(B)** Dotplot of GO biological process analysis of 2,561 protein-coding Up DEGs. **(C)** A category netplot composed of the top three downregulated biological processes from **(A)** and the individual DEGs within each ontology. **(D)** A category netplot composed of the top three upregulated biological processes from **(B)** and the individual DEGs within each ontology.

After observing global transcriptional dysregulation with 1,474 downregulated genes and 3077 upregulated genes, we sought to parse out what direction particular biological processes are due to *Taf4b* ablation. Of the 1,259 downregulated genes that were protein-coding, the top three biological process gene ontology categories were cilium movement, negative regulation of the immune system process, and cell killing (Figures 3A,C). From the upregulated genes, 2,561 were protein-coding, and we saw that the biological process categories are primarily related to cell cycle, DNA repair, and covalent chromatin modification (Figures 3B,D). Surprisingly, there were almost twice as many upregulated genes than downregulated genes in the absence of TAF4b, many of which encoded critical components of the cell cycle and chromatin regulatory machinery.

### *Taf4b*-deficient ProSpg are delayed in their entry into quiescence

**FIGURE 4:**
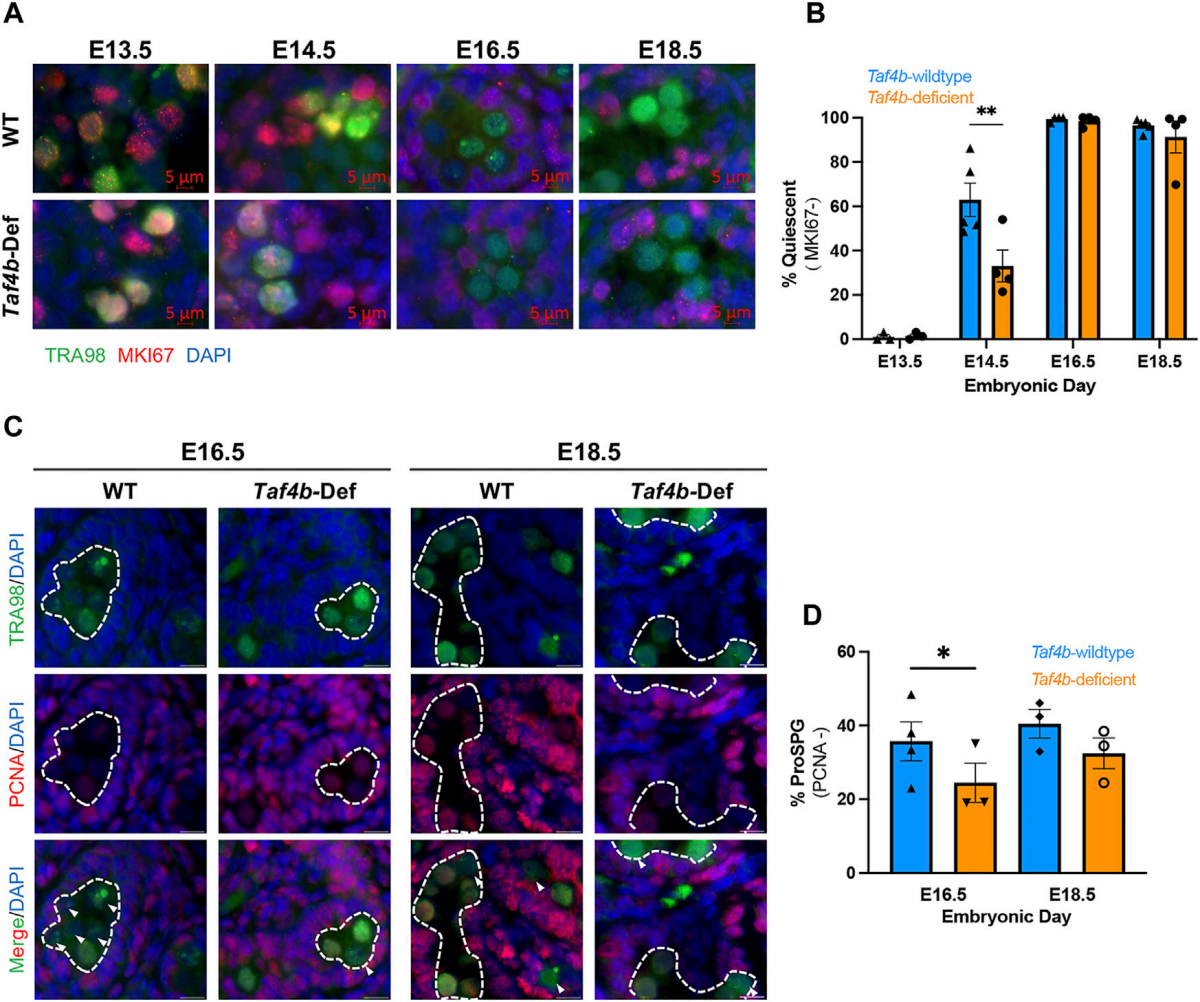
*Taf4b*-deficient ProSpg have increased MKI67 at E14.5. **(A)** Immunofluorescence of E13.5-E18.5 testis sections comparing *Taf4b*-wildtype (TAF4b +/+) to *Taf4b*-deficient ProSpg (TAF4b −/−). Antibodies for TRA98 (green), MKI67 (red) were used, and DAPI labels DNA. **(B)** Quantification of MKI67^-^/TRA98^+^ cells in *Taf4b*-wildtype (blue) and *Taf4b*-deficient (orange) testis sections. *Taf4b*-deficient ProSpg have significantly fewer MKI67^-^ ProSpg than wildtype at E14.5 (n = 3-5 mice, ** = p-adj< 0.01 as determined by two-way ANOVA). **(C)** Immunofluorescence of E16.5-E18.5 testis sections comparing *Taf4b*-wildtype (TAF4b +/+) to *Taf4b*-deficient ProSpg (TAF4b −/−). Antibodies for TRA98 (green), PCNA (red) were used, and DAPI (blue) labels DNA. **(D)** Quantification of PCNA^−^/TRA98^+^ cells in *Taf4b*-wildtype (blue) and *Taf4b*-deficient (orange) testis sections. *Taf4b*-deficient ProSpg have significantly fewer PCNA^−^ ProSpg than wildtype at E16.5 (n = 3-4 mice, * = *p*-value< 0.05 as determined by paired t-test).

Mitotic ProSpg enter a characteristic period of quiescence between E13.5 and E16.5. Based upon our scRNA-seq and bulk RNA-seq data, we hypothesized that TAF4b modulated some aspect of ProSpg quiescence. We performed immunofluorescence on *Taf4b*-wildtype and *Taf4b*-deficient testis sections to detect TRA98 (a germ cell marker) and MKi67 (a marker of cell proliferation) from E13.5 to E18.5 (Grive et al., 2014; Sun and Kaufman, 2018). Representative images of each genotype and time point are shown (Figure 4A), as well as the quantification of the MKi67^-^ quiescent wildtype and *Taf4b*-deficient ProSpg (indicated by the TRA98-only signal) at each timepoint (Figure 4B). At E13.5, the majority of *Taf4b*-deficient M ProSpg were still proliferating as indicated by the nuclear localization of MKi67 (Figure 4A). At E14.5, there is a significant reduction of ProSpg entering quiescence in the *Taf4b*-deficient compared to wildtype controls (Figure 4B). Interestingly, the percentage of quiescent cells at both E16.5 to E18.5 did not differ significantly between controls and *Taf4b-*deficient Pro-Spg. These data indicate that although *Taf4b*-defcient ProSpg can largely achieve quiescence, they struggle with its timely entry and this transition. We also compared staining for the proliferating cell nuclear antigen (PCNA), which is a DNA replication and repair protein, across matched *Taf4b*-wildtype and *Taf4b*-deficient testis sections (Figure 4C). At E16.5, there is a significant increase in the percent of *Taf4b*-deficient ProSpg expressing PCNA compared to matched wildtype controls and this difference is non-significant at E18.5 (Figure 4D). Taken together, these data indicate that *Taf4b*-deficient ProSpg are altered in their timing of ProSpg quiescence entry.

### CUT&RUN identifies direct targets of TAF4b in E16.5 ProSpg

**FIGURE 5:**
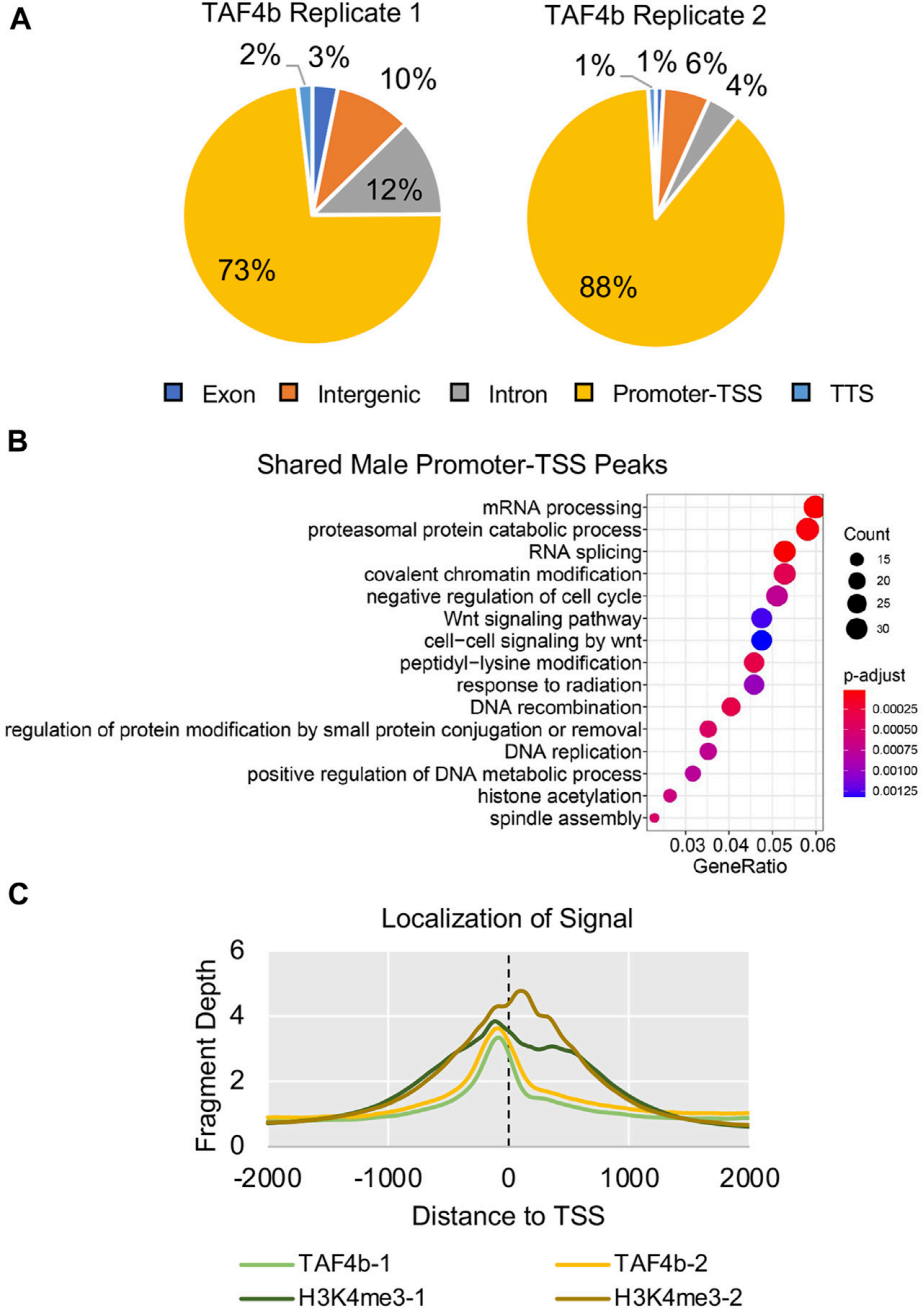
Genome-wide Occupancy of H3K4me3 and TAF4b in E16.5 ProSpg. CUT& RUN of E16.5 ProSpg using antibodies for H3K4me3 and TAF4b. **(A)** Pie charts of TAF4b peak locations across the E16.5 ProSpg genome in two independent CUT&RUN replicates. **(B)** GO biological process dotplot for the shared CUT&RUN peaks categorized as “promoter-TSS”. **(C)** Average enrichment of TAF4b and H3K4me3 signal near TSSs (dotted line) for each replicate.

To distinguish which DEGs identified in our E16.5 bulk RNA-seq experiment were direct targets of TAF4b, we performed Cleavage Under Targets and Release Using Nuclease (CUT&RUN), a genome mapping technique to identify enrichment of specific proteins and histone modifications in the genome (Skene and Henikoff, 2017; Hainer and Fazzio, 2019). We isolated two replicates of wildtype *Oct4*-eGFP E16.5 male germ cells using FACS and examined the genomic localization of TAF4b. H3K4me3 served as a positive control and marked active promoters, and IgG served as a negative control. CUT&RUN data analysis using the program Homer identified 64,891 H3K4me3 peaks and 7,861 TAF4b peaks in Replicate 1 and 1,730 H3K4me3 peaks and 848 TAF4b peaks in Replicate 2. The differences in the number of peaks called can be attributed to stochastic differences in sample preparation and starting material. However, it is necessary to note that these differences did not affect the assay’s specificity because we still see the same top motifs and the greater than 80% overlap between each replicate. We also found that 73% and 88% of TAF4b peaks were classified as localizing to promoters/transcription start site (“promoter-TSS”) for Replicate 1 and 2, respectively (Figure 5A). Importantly, 617 TAF4b peaks overlapped between the two replicates allowing us to perform GO analysis of the shared TAF4b-bound gene promoter-TSSs between the two replicates. These top five categories include mRNA processing, proteasomal degredation, RNA splicing, covalent chromatin modification, and negative regulation of the cell cycle (Figure 5B). When plotting the enrichment profile of TAF4b relative to TSSs, we found the highest TAF4b enrichment was located just upstream of the TSS (Figure 5C). This initial chromatin mapping of TAF4b indicates that the majority of the binding sites for TAF4b are just upstream of the TSS which is consistent with where TFIID is known to bind to core promoters.

**FIGURE 6:**
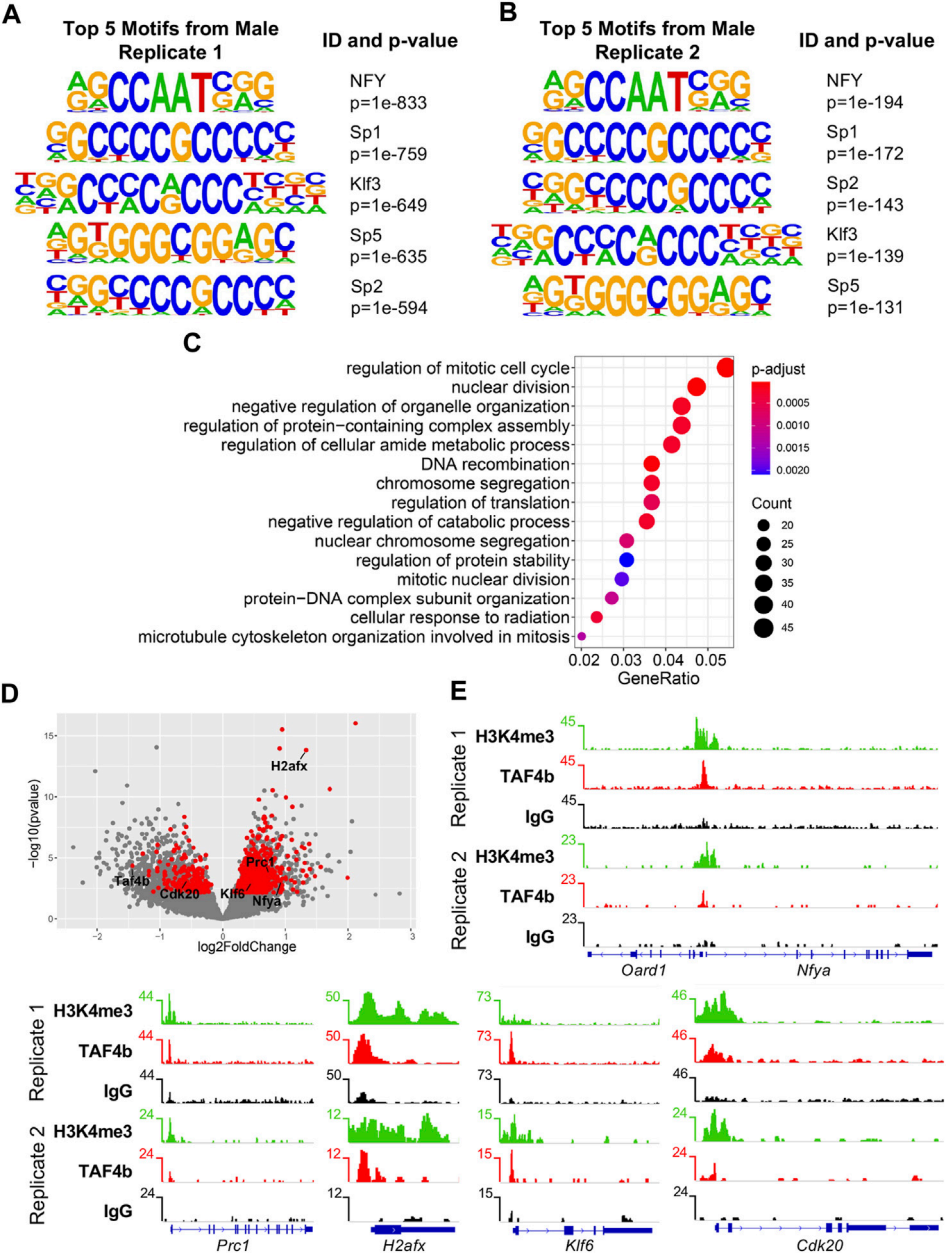
CUT&RUN and RNA-seq integration identify TAF4b bound and modulated cell cycle genes. **(A)** Top five TAF4b motifs from “promoter-TSS” peaks in Top five TAF4b motifs from “promoter-TSS” peaks in male Replicate 1. **(B)** Top five TAF4b motifs from “promoter-TSS” peaks in male Replicate 2. **(C)** Biological process GO dotplot of the 876 genes that are DEGs and had a TAF4b promoter-TSS peak in at least one of the two germ cell samples. **(D)** Volcano plot of the 876 DEGs that had at least one TAF4b promoter peak (red dots). **(E)** Gene track of *Oard1* and *Nfya*, in which *Nfya* was a DEG that had a TAF4b promoter-TSS called in both replicates. Gene tracks of *Prc1*, *H2afx*, *Klf6*, and *Cdk20*, which were DEGs (labeled red dots in D) that had a TAF4b promoter-TSS called in both replicates.

We then explored the motifs associated with TAF4b-enriched peaks within our CUT&RUN data and found consistent enrichment of CCAAT and GC motifs, which are associated with the NFY complex and the Sp/Klf transcription factor family, respectively (Figures 6A,B) (Suske, 2017). Importantly, these motifs were shared between the two CUT&RUN replicates. We then compared DEGs from our RNA-seq experiment to direct targets of TAF4b from the CUT&RUN to identify a subset of genes directly modulated by TAF4b at E16.5. When comparing our DEGs to the “promoter-TSS” peaks of TAF4b, we found 876 DEGs that had at least one peak near their TSS. GO analysis of these 876 TAF4b bound and differentially expressed genes reveals that the most notable biological process affected is “regulation of the mitotic cell cycle” (Figure 6C). A volcano plot of these TAF4b-bound E16.5 DEGs revealed far more upregulated DEGs (674 genes) than downregulated DEGs (202 genes) in *Taf4b*-deficient ProSpg (Figure 6D). As examples of TAF4b-bound DEGs, we present TSS gene tracks for Nuclear Transcription Factor Y Subunit Alpha (*Nfya)*, Protein Regulator of cytokinesis 1 (*Prc1)*, H2A histone family member X (*H2afx)*, Kruppel-like factor 6 (*Klf6)*, and Cyclin Dependent Kinase 20 (*Cdk20)*, with TAF4b and H3K4me3 enrichment compared with control IgG (Figure 6E). *Nfya* is a component of the NFY transcription factor complex and interestingly TAF4b peaks were located between the TSS of *Nfya* and another gene *Oard1* which was not a DEG. *Prc1* encodes a microtubule-binding protein that plays a role in mitosis and binds to *Plk1* another critical mitosis gene (Hernández-Ortega et al., 2019). *H2afx* encodes an essential DNA damage response protein and has been previously found to be increased in *Taf4b*-deficient oocytes (Grive et al., 2016). *Klf6* encodes a transcription factor that plays a role in several developmental processes such as cell proliferation and differentiation (Hu et al., 2021). *Cdk20,* decreased in *Taf4b-*deficient ProSpg, encodes a positive regulator of the cell cycle by activating CDK2 and Cyclin D (Lai et al., 2020). A protein-protein interaction network of these 876 TAF4b-bound DEGs revealed *Cdk1* and other cell cycle-related genes as a major group of associated genes and interestingly the entire NFY complex (encoded by *Nfya*, *Nfyb*, and *Nfyc*) was present (Supplementary Figure S1).

### TAF4b-bound and differentially expressed ProSpg gene promoters are enriched for related specificity protein and kruppel-like family (SP/KLF) of transcription factor binding motifs

**FIGURE 7:**
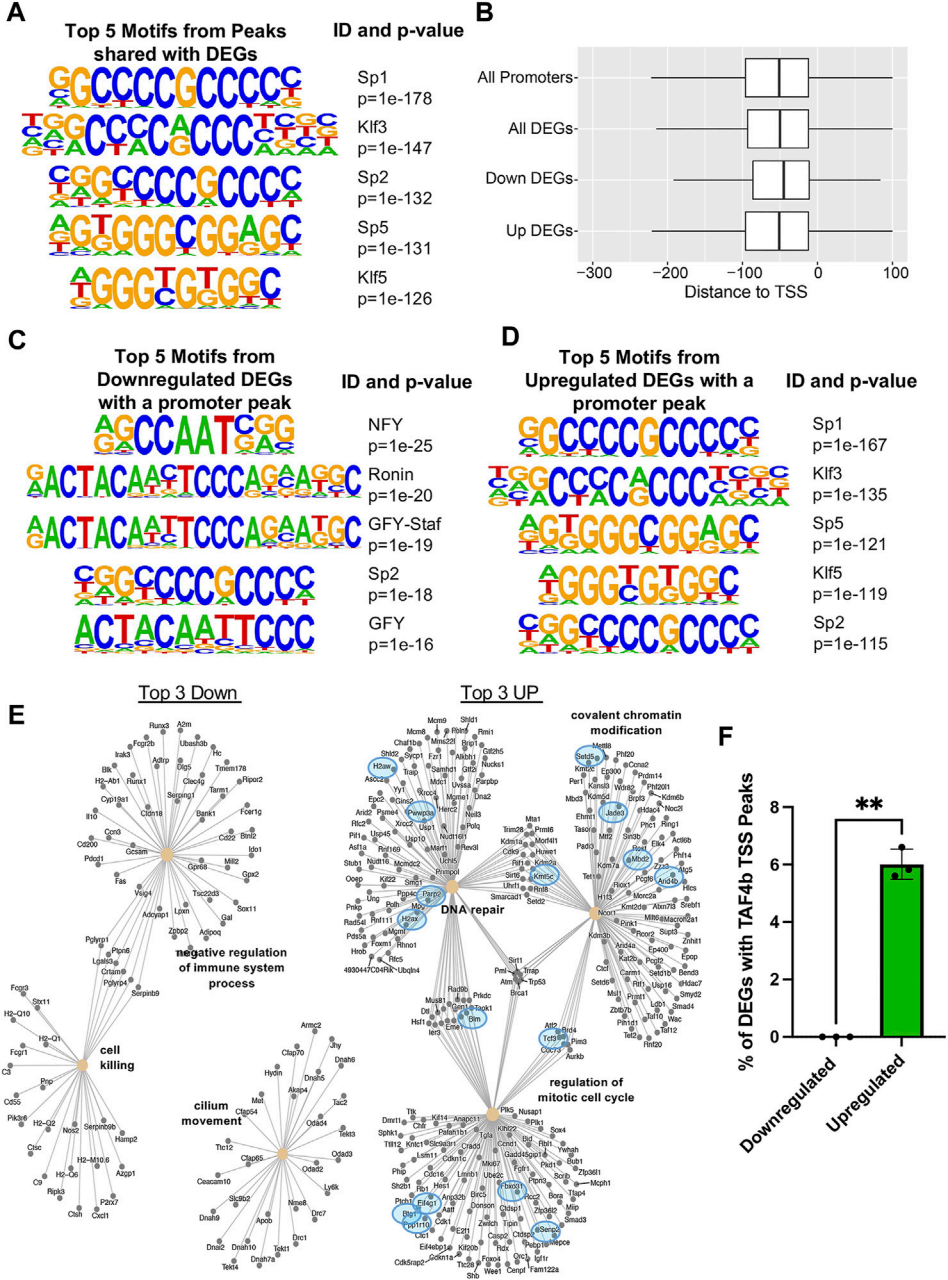
Sp/Klf motif enrichment at cell cycle and chromatin regulating TAF4b bound gene promoters. **(A)** Top five motifs enriched at TAF4b “promoter-TSS” peaks for genes that were also DEGs, the promoter ID, and the associated *p*-value. **(B)** Boxplots (no outliers included) of promoter TAF4b peaks relative to the TSS with a median of −53 bp, all TAF4b “promoter-TSS” peaks for genes that were also DEGs with a median of −51 bp, TAF4b “promoter-TSS” peaks for genes that were only Downregulated DEGs with a median of −49 bp and TAF4b “promoter-TSS” peaks for genes that were only Upregulated DEGs with a median of −53 bp. **(C)** Top five TAF4b associated binding motifs from “promoter-TSS” peaks in Down DEGs and **(D)** Up DEGs. **(E)** Category netplots composed of the top three downregulated (left) and upregulated (right) biological processes from (Figures 3C,D) along with DEGs that contained TAF4b peaks at their TSS (blue circles). **(F)** Comparison of TAF4b target DEG percentages from top three upregulated and downregulated GO categories. Significance determined by Welch’s t-test, ** = *p*-value <0.01.

When integrating our CUT&RUN “promoter-TSS” peaks with TAF4b DEGs, we again found an enrichment for SP/KLF transcription factor binding motifs (Figure 7A). However when we looked at the integrated data we no longer observed NFY in the top five overall binding motifs. The surprising absence of NFY sites in this analysis prompted us to further parse these data into TAF4b peaks whose levels of mRNA decreased (Downregulated DEGs) or increased (Upregulated DEGs) in TAF4b-deficient ProSpg. We first looked at the occupancy of TAF4b and noted that it centered around the −50 bp region upstream of the TSS, further supporting a role for TAF4b in core promoter recognition and transcription initiation. It was also observed that there was a slight difference in the median being approximately 5 bases futher upsteam in the Up DEGs as compared to the Down DEGs (Figure 7B). Interestingly, we discovered that NFY is actually the top Down DEG motif while Sp1 is the top Up DEG motif (Figures 7C,D). The differences in top motifs between decreased and increased mRNA levels may allude to the multiple modes of transcriptional modulation by TAF4b. As follow-up, we mapped TAF4b targets to the GO analysis from bulk RNA-seq to determine which GO categories were directly modulated. Most notable in this analysis were the categories of cell cycle regulation, covalent chromatin modification, and DNA repair which had increased mRNA expression levels in the absence of TAF4b and also contained 7 genes that had TAF4b-bound promoters (Figure 7E). Finally, we quantified the overall fraction of CUT&RUN peaks that were also DEGs and found significantly more mRNAs that were increased in the absence of TAF4b to contain a TAF4b-bound promoter (Figure 7F). Overall, these data suggest that TAF4b specifically controls the levels of cell cycle and chromatin regulating gene expression programs required for ProSpg to properly navigate the transition from cycling to quiescence during embryonic mammalian testes development.

## Discussion

Soon after sex determination in mammals, embryonic female and male germ cells receive distinct signals to initiate a sex-specific germ cell differentiation program (Capel, 2019). However, it is unclear how these newly defined germ cells integrate external signals to execute precise and sex-specific gene expression programs and cell cycle states required for germ cell development. We have discovered that the embryonic germline-enriched TFIID subunit, TAF4b, is required for male and female germ cell development and maintenance in mice. Here, we identify many ProSpg genes whose expression are affected by the loss of TAF4b and whose core promoter regions display TAF4b occupancy just upstream of the TSS. TAF4b enrichment at ProSpg core promoters correlates with the binding sites for Sp1 and NFY, two well-characterized and ubiquitous promoter-proximal and sequence-specific transcription factors. The integration of these high throughput genomic sequencing and mapping data indicate that TAF4b fine tunes male-specific cell cycle gene expression required for the timely entry of ProSpg into quiescence via complex and unexpected mechanisms.

Several notable regulators of post-transcriptional gene expression play similar developmental functions as TAF4b when ProSpg transition towards quiescence. The most notable is the RNA-binding protein DND1 which displays intriguing parallels with the functions of TAF4b shown here. DND1 deficiency in mice leads to defects of prospermatogonial entry into quiescence and increased germ cell loss (Cook et al., 2011). However, the Ter mutation in the *Dnd1* gene on the 129 genetic background strikingly results in spontaneous teratoma formation at E16.5 (Cook et al., 2011). While *Taf4b*-deficient testes display similar kinetics in male germ cell loss and fertility disruption, our *Taf4b*-deficiency presented here is on a C57Bl/6 background where we might not expect to see these teratomas (Falender et al., 2005). Remarkably, TAF4b and DND1 share common regulation of mitotic cell cycle and chromatin modification gene expression, suggesting they are two distinct modes of regulating a common ProSpg quiescence and survival program ((Ruthig et al., 2019), this study). A second RNA-binding protein, NANOS2, is also responsible for ProSpg development and has been recently shown to be bound to and work with DND1 in regulating specific RNA loading into the CNOT deadenylase complex (Suzuki et al., 2016; Ruthig et al., 2019). Interestingly, *Nanos2* is an upregulated DEG, and *Dnd1* is a downregulated DEG in E16.5 *Taf4b*-deficient ProSpg. The developmental similarities of perturbed quiescence, increased germ cell loss, and infertility in *Nanos2, Dnd1*, and *Taf4b* mouse mutants suggest that regulating the gene expression of prospermatogonia transitions is critical to properly set up the future spermatogonia that arise from this lineage in the postnatal testis. The dysregulation of *Nanos2* and *Dnd1* in the absence of TAF4b also indicates cross-talk between these crucial gene expression regulators and the sensitivity of germline development.

There are also several key transcription factors that direct cell cycle progression and transcription during prospermatogonial development. The master regulator of the G1 phase of the cell cycle, RB1, is required for the timely mitotic arrest of early prospermatogonia (Spiller et al., 2010). A recent germ cell-specific knockout approach for RB1 linked this cell cycle defect to disrupting the metabolic nature of these developing prospermatogonia and their corresponding ability to become SSCs (Du et al., 2021). While the timing of RB1 and TAF4b functions in promoting quiescence are similar, RB1 deficiency leads to much more severe cell cycle defects than *Taf4b*-deficiency, suggesting they play more complementary roles in SSC development (Du et al., 2021). RHOX10 is a homeodomain family sequence-specific DNA-binding protein found in the reproductive homeobox cluster of the X chromosome and is required for prospermatogonial and SSC development. A recent genomic study using similar tools implemented here uncovered a network of transcription factors that are direct targets of RHOX10, most notably DMRT1 and ZBTB16 (Tan et al., 2021). While there are minor differences in the timing and cells used in these two studies, Tan et al., 2021 uncovered a critical CCAAT box bound by RHOX10 in the Dmrt1 promoter and predicted it to be a binding site for the NFY transcription factor, which was the most correlated binding site at TAF4b bound core promoters (Tan et al., 2021). It will be interesting to determine how these transcriptional regulators work together and separately to promote prospermatogonial development required for the proper establishment of SSCs and longterm mammalian spermatogenesis.

The precise molecular mechanism of TAF4b in promoting ProSpg transitions remains unknown, but several interesting new twists are revealed here. TFIID was first discovered as a ubiquitous multiprotein core promoter binding and coactivator complex consisting of the TATA-box binding protein and 14 TBP-associated factors (TAFs) required for Sp1-dependent transcriptional activation *in vitro* (Pugh, 1990). However, determining Sp1 as the top binding motif at the cell cycle and chromatin modification gene promoters bound by TAF4b in ProSpg was surprising, given this unique cell type-specificity. Moreover, mRNA expression levels of these gene programs primarily increase in the absence of TAF4b, suggesting TAF4b plays some role in limiting their expression as ProSpg transitions to quiescence. Future studies will determine whether TAF4b works in a chromatin modification capacity along with its canonical function in the TFIID complex. As TAF4b regulates chromatin modification genes, we also plan on determining if changes in chromatin accessibility are a strong driver of our observed gene expression changes. This non-canonical role of TAF4b may contribute to proper silencing and timely repression of specific genes during spermatogenesis. We also know now that other members of the TFIID complex have been found in chromatin-modifying complexes like that of the Spt/Ada/Gcn5 acetyltransferase (SAGA) complex. Members such as TAF5L, TAF6L, and TAF9B are just some with documented dual activity (Soffers and Workman, 2020). Another possibility is that as later T2 ProSpg return to proliferation quickly after birth, TAF4b helps mark the cell cycle and chromatin modifying genes for future activation during later stages of postnatal germ cell expansion. Lastly, the TATA-box that marks a subset of TFIID-dependent promoters is usually found between −25 bp and −30 bp upstream of the TSS, and our peaks of TAF4b binding center around the −50 bp region suggesting that TAF4b might extend the upstream boundary of the TFIID footprint at the core promoter by 20 bp (Sloutskin et al., 2021). Future studies of ProSpg core promoter architecture and TAF4b′s transcriptional mechanism with individual SP/KlF and NFY family members will be required to understand these novel aspects of ProSpg transcription regulation.

While we focus here on TAF4b’s role in promoting the early development of the male germline, we have recently reported that at identical embryonic time points in ovarian development, TAF4b plays a similar critical role in early oocyte differentiation (Gura et al., 2022). As female and male embryonic germ cells express unique sets of genes and navigate different biological processes, i.e., meiotic initiation vs. mitotic arrest, it is surprising that the same transcription factor would have distinct functions in the early life of these future gametes. We propose that one common function of TAF4b, and its associated regulatory complex, is to promote the embryonic germ cell identity and survival after PGC specification, analogous to the germ cell licensing function of DAZL (Gill et al., 2011). In addition, TAF4b has likely evolved sex-specific functions that allow it to promote context-specific transcription events in both male and female developing germ cells. One commonality of these two populations is that even though they enter meiosis at different times, they both exit the mitotic cell cycle at the end of PGC development, and TAF4b, along with many other unknown regulatory proteins, helps direct these transitions. Finally, genome-wide epigenetic modifications are erased in both female and male germ cell populations at the end of PGC development. In the context of a more open genome, TAF4b and its related regulators could help maintain the appropriate chromatin environment for controlling transcription-coupled DNA repair mechanisms unique and critical to the germline genome of both sexes and, ultimately, the next-generation.

## Data Availability

The male mouse E16.5 RNA-sequencing data are available from NCBI GEO under accession number GSE188351. The male mouse E16.5 CUT&RUN sequencing data are available from NCBI GEO under accession number GSE188701. The sequencing datasets accessed in this research are from the follow accession numbers: the scRNA-seq mouse data from E12.5 to P7 male sorted Oct4-eGFP gonads used for Figure 1 was obtained through NCBI GEO: Q9 GSE119045, GSE124904, and GSE130593.

## Supplementary material

SUPPLEMENTARY TABLE S1 Cell numbers for RNA-seq samples.

SUPPLEMENTARY TABLE S2 Output for E16.5 ProSpg RNA-seq.

SUPPLEMENTARY TABLE S3 TAF4b and H3K4me3 CUT&RUN in E16.5 ProSpg.

SUPPLEMENTARY FIGURE S1 Protein-protein interactions of TAF4b bound DEGs in E16.5 ProSpg.
