# Supplementary figures and images for: Transcription and chromatin regulation by TAF4b during cellular quiescence of developing prospermatogonia
Source: Front Cell Dev Biol. 2023 Oct 12;11:1270408. doi: 10.3389/fcell.2023.1270408 (PMC10600471; doi:10.3389/fcell.2023.1270408)

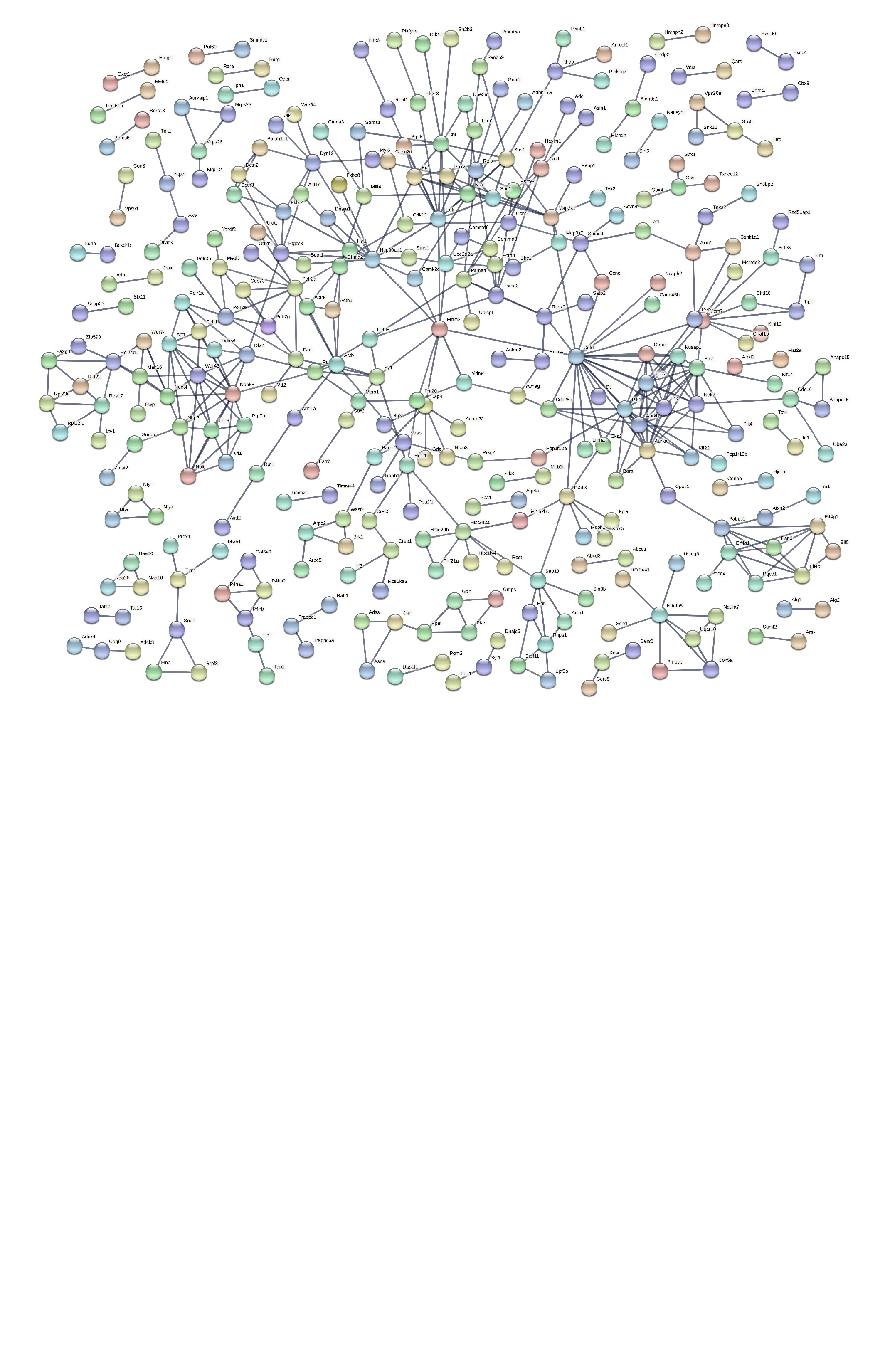

Supplement: Supplementary file 1 [file Presentation1.zip › Gura et al., Supplemental Files/Supplementary Figure 1.tif]

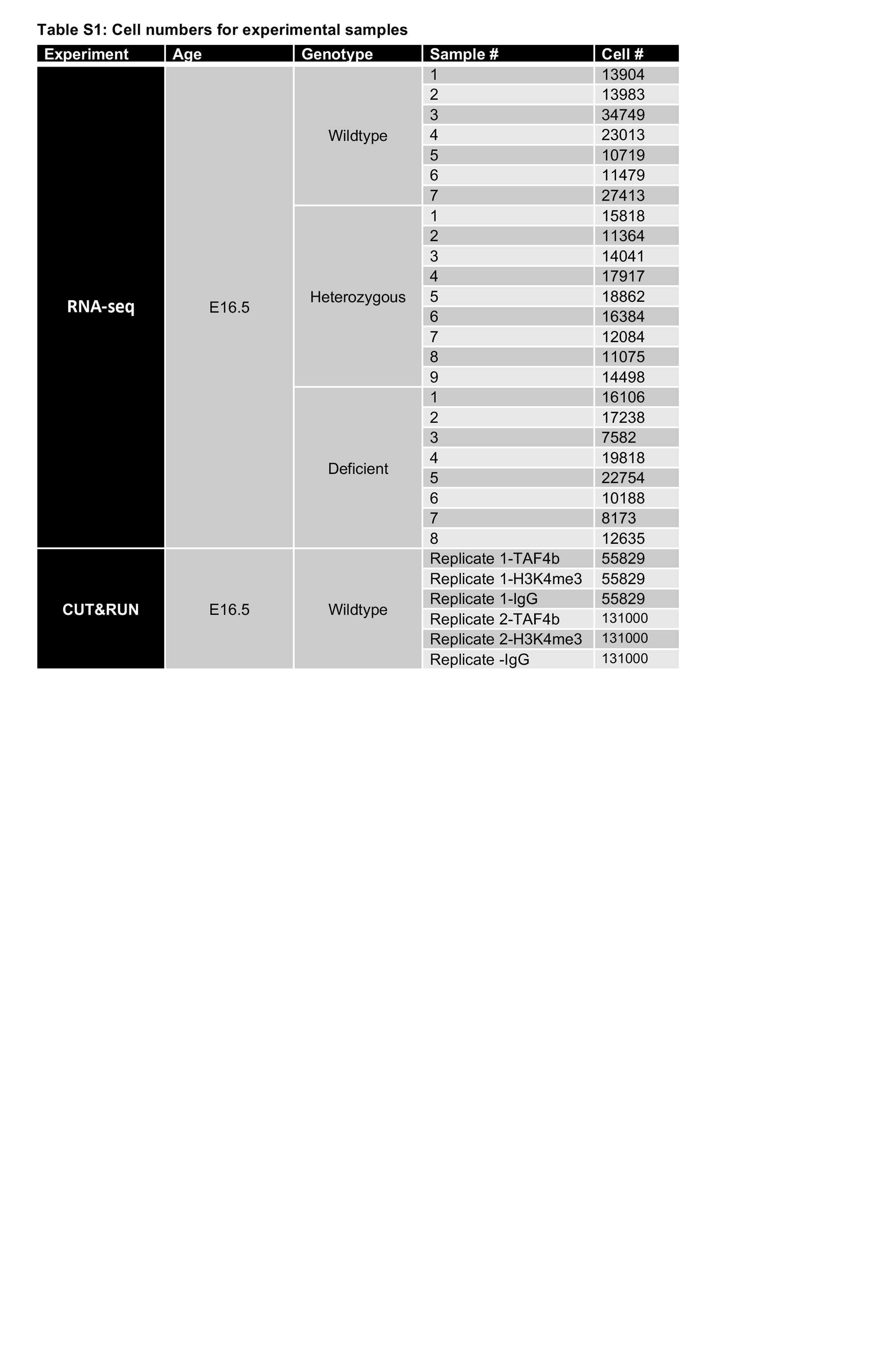

Supplement: Supplementary file 1 [file Presentation1.zip › Gura et al., Supplemental Files/Supplementary Table 1.tif]
